# Effect of liraglutide on expression of inflammatory genes in type 2 diabetes

**DOI:** 10.1038/s41598-021-97967-0

**Published:** 2021-09-17

**Authors:** Emilie H. Zobel, Rasmus S. Ripa, Bernt J. von Scholten, Viktor Rotbain Curovic, Andreas Kjaer, Tine W. Hansen, Peter Rossing, Joachim Størling

**Affiliations:** 1 Steno Diabetes Center Copenhagen, Niels Steensens Vej 2, 2820 Gentofte, Denmark; 2 Department of Clinical Physiology, Nuclear Medicine & PET and Cluster for Molecular Imaging, Rigshospitalet, University of Copenhagen, Copenhagen, Denmark; 3 Novo Nordisk A/S, Søborg, Denmark; 4 Department of Clinical Medicine, University of Copenhagen, Copenhagen, Denmark; 5 Department of Biomedical Sciences, University of Copenhagen, Copenhagen, Denmark

**Keywords:** Endocrinology, Diabetes

## Abstract

Anti-inflammatory effects of glucagon-like peptide 1 receptor agonist (GLP-1 RA) treatment in T2D may contribute to the cardiovascular benefits observed with GLP-1 RAs in outcome studies. We investigated if the GLP-1 RA liraglutide exerts anti-inflammatory effects through modulation of inflammatory gene expression in peripheral blood mononuclear cells (PBMCs). From 54 participants of a double-blinded trial where individuals with type 2 diabetes (T2D) were randomized to liraglutide (1.8 mg/day) or placebo for 26 weeks, a sub-study was performed in which PBMCs were extracted from fresh blood at study start and at end-of-treatment. The expression of selected inflammatory genes in PBMCs were measured by quantitative real-time polymerase chain reaction (PCR). Moreover, the expression of the *GLP-1 receptor (GLP1R)* was examined in a subset (n = 40) of the PBMC samples. The human monocytic cell line THP-1 was used for in vitro GLP-1 exposure experiments. The expression of tumor necrosis factor-α (*TNFA*) (p = 0.004) and interleukin-1β (*IL1B*) was downregulated (p = 0.046) in the liraglutide-treated group (n = 31), and unchanged in the placebo group (n = 21, p ≥ 0.11), with no significant differences between the two groups (p ≥ 0.67). The expression of interferon-γ (*IFNG*) and cluster of differentiation 163 (*CD163*) were upregulated in both groups (p ≤ 0.006) with no differences between groups (p ≥ 0.47). C–C Motif Chemokine Ligand 5 (*CCL5*) was upregulated in the liraglutide-treated group (p = 0.002) and unchanged in the placebo group (p = 0.14), with no significant difference between groups (p = 0.36). Intercellular adhesion molecule 1 (*ICAM1*) was unchanged in both groups (p ≥ 0.43). *GLP1R* expression in the PBMCs was undetectable. In vitro experiments showed no effect of GLP-1 treatment on inflammatory gene expression in THP-1 cells. *GLP1R* expression in THP-1 cells was not detectable. In summary, we observed a discrete modulatory effect of liraglutide on the expression of inflammatory genes in PBMCs. The lack of evidence for *GLP1R* expression in PBMCs and THP-1 cells suggests that possible effects of liraglutide on the PBMCs’ gene expression are most likely indirect. Further investigations are needed to establish the anti-inflammatory potential of GLP-1 RAs.

## Introduction

Low grade inflammation contributes to the pathophysiology of type 2 diabetes (T2D) and may play an important role in the development of diabetic complications, including cardiovascular disease (CVD)^1,2^. Treatment with glucagon-like peptide-1 receptor agonists (GLP-1 RAs) reduces the risk of CVD in T2D patients with a history of CVD or high risk for CVD^3–6^. It has been hypothesized that the underlying mechanism of this beneficial GLP-1 RA effect is an preventive effect of GLP-1 RAs on atherosclerotic plaque formation^7^ and/or anti-inflammatory actions of GLP-1 RAs. Hence, clinical studies have suggested that GLP-1 RA treatment reduces small dense low-density lipoproteins^8^, carotid-intima media thickness^8,9^, specific microRNAs involved in vascular inflammation and cardiovascular metabolism^10^, oxidative stress^11^ and circulating inflammatory markers^12–14^. The inflammatory markers reduced by GLP-1 RA in clinical studies in humans, include circulating plasma levels of tumor necrosis factor (TNF)-α^15^, interleukin (IL)-1β, IL-6 and cluster of differentiation 163 (CD163)^16^. In a large randomized trial, GLP-1 RA treatment reduced the plasma level of high-sensitivity C-reactive protein (hsCRP) by approximately 35%^17^. However, we are far from fully understanding the anti-inflammatory effects of GLP-1 RAs including which tissue(s) is targeted by GLP-1 RAs leading to reduced circulating inflammation.

Peripheral blood mononuclear cells (PBMCs) consist of lymphocytes and monocytes, which are profoundly engaged in the production of inflammatory factors such as cytokines. PBMCs constitute an accessible human tissue and therefore offer an opportunity to investigate if GLP-1 RA treatment alters the expression of genes encoding inflammatory factors. Two small clinical studies previously indicated that GLP-1 RA treatment affects inflammatory gene expression in PBMCs^12,18^.

We recently performed a randomized double-blind placebo-controlled clinical trial with the primary aim of investigating the effect of 26 weeks treatment with the GLP-1 RA liraglutide on arterial inflammation assessed by Positron Emission Tomography (PET)/ Computed Tomography (CT)^19^. Here we report the results of a sub-study investigating the expression of selected inflammatory genes in PBMCs at start and end-of-treatment. In parallel in vitro experiments, we investigated if GLP-1 RA treatment modified the gene expression of inflammatory genes using the human monocyte THP-1 cell line.

## Materials and methods

### Study design of the randomized controlled clinical trial

A sub-study of a double-blinded trial where persons with T2D were randomized to liraglutide up to 1.8 mg on top of standard of care or placebo on top of standard of care once daily for 26 weeks. The main trial included 102 patients with the primary aim of evaluating liraglutide’s effect on arterial inflammation. The trial included patients with T2D above 50 years of age, with an HbA1c ≥ 48 mmol/mol (6.5%) and eGFR ≥ 30 mL/min/1.73 m^2^. Participants were on stable glucose- and cholesterol-lowering treatment for a minimum of 4 weeks prior to inclusion. Treatment with dipeptidyl peptidase 4 inhibitors or GLP-1 RAs were not allowed 90 days prior to screening. Description of the main trial including detailed in- and exclusion criteria has previously been published^19^. The primary end-point of the trial was change in vascular uptake of ^18^F-fluorodeoxyglucose (FDG) and was unchanged^19^. Based on pharmacokinetic profile studies, we expected that liraglutide dosing between 0.6 and 1.8 mg once daily would result in a serum concentration of liraglutide of approximately 5–40 nmol/L^20^.

In the current study, we report the results of a sub-study where the expression of inflammatory cytokines was evaluated before and at end-of-treatment in 54 (53%) of the study participants. The number of included participants in this sub-study was conditioned by the feasibility of PBMC isolation from blood samples at time of scheduled visits in the main trial, and inclusion was random.

Local ethics committee (Committee E, Region H, Denmark, H-16044546) and the Danish Medicines Agency (2016110109) approved the trial. The trial was conducted in compliance with the principles of the Declaration of Helsinki. Trial registration: ClinicalTrials.gov (NCT03449654) and EU Clinical Trials Register (2016-001523-31). All participants provided written informed consent.

### PBMC isolation

Peripheral blood mononuclear cells were prepared from fresh blood samples collected in Ethylenediaminetetraacetic acid (EDTA) tubes using SepMate™ PBMC Isolation Tubes and Lymphoprep™ according to the manufacturer´s protocol (Stemcell Technologies). Briefly, 8 ml of anticoagulated blood was mixed with 8 ml of phosphate-buffered saline (PBS) which was then gently added to a SepMate™ tube containing 15 ml of Lymphoprep™. Following centrifugation for 10 min at 1200 × g at room temperature, the top layer containing the plasma and PBMCs was poured into a 50 ml tube. Following centrifugation for 5 min at 300 × *g*, the supernatant was discarded and the PBMC pellet was washed once with 5 ml PBS. After pelleting by centrifugation, the PBMCs were resuspended in 100 μl PBS to which 500 μl RNAlater (Qiagen) was added. The total volume of 600 μl was divided equally into three Eppendorf tubes and stored at − 80 °C until further analysis.

### THP-1 cell experimentation

Human monocytic THP-1 cells were grown in RPMI1640 medium containing 4.5 g/L glucose and 10% fetal bovine serum. Cells were passaged twice weekly. Cells seeded in duplicates were differentiated into macrophages by exposure to 100 nM phorbol 12-myristate 13-acetate for 3 days after which the cells were left untreated or exposed to 5 ng/mL lipopolysaccharide (LPS) for 3 h in the presence or absence of 2.5 nM recombinant GLP-1 (amino acids 7–36) (R&D Systems). GLP-1 (7–36) and liraglutide has been shown to have very similar in vitro effects^21,22^. The dose of 2.5 nM was chosen based on previous studies showing that GLP-1 in the low nM range exerts maximum or near maximum receptor binding and/or cellular effects^23–25^. After stimulation, cells were washed in ice-cold Hanks’ balanced salt solution (Gibco) before being lysed in RLT buffer (Qiagen).

### Gene expression

RNA was extracted from PBMCs by the Maxwell® 16 Tissue LEV Total RNA Purification Kit using a Maxwell™ 16 purification instrument according to the manufacturer´s protocol (Promega). RNA from THP-1 cells was extracted using miRNeasy mini kit spin columns (QIAGEN). cDNA was prepared using the iScript™ cDNA Synthesis Kit (Bio-Rad). Gene expression was quantified by real-time PCR using TaqMan assays (Applied Biosystems) on a CFX384 C1000 Termal Cycler (Biorad). Gene expression was normalized to that of actin B (*ACTB*). The relative expression levels were calculated by the ΔCt method.

### Statistical analysis

Power calculation was done for the main trial with a primary PET/CT endpoint, and not for the present sub-study^19^. We aimed at including as many participants as possible from the main trial and ended up with 31 participants treated with liraglutide and 23 treated with placebo**.**

Normally distributed data are presented as mean (standard deviation (SD)). Diabetes duration, urinary albumin creatinine ratio and hsCRP were log2 transformed in all analyses and presented as median [Interquartile range (IQR)]. Categorical variables are presented as total numbers (percent). Differences in clinical characteristics at baseline were tested using un-paired t-test, χ^2^ test, or Fisher’s exact test as appropriate. We applied paired t-test to compare baseline and end-of-treatment values within the liraglutide and placebo treated group. Unpaired t-test was used to compare the change from baseline to end-of-treatment between the liraglutide and placebo treated group. Distributions were checked for normality before using paired and un-paired t-tests. Statistical analyses were performed using SAS software (version 9.4; SAS Institute, NC) and two-sided p-values < 0.05 were considered statistically significant.

## Results

### Baseline characteristics of study participants

This sub-study included 54 participants from which PBMCs were isolated and RNA extracted to determine the expression of various inflammatory genes selected based on literature searches at baseline and after 26 weeks of treatment with liraglutide/placebo. The mean age (SD) was 66.8 (8.4) years, HbA_1c_ was 57.4 (9.7) mmol/mol, median (IQR) hsCRP was 2.3 (0.97–4.7) mg/L and 11 (20.4%) had a history of CVD (Table 1). Baseline characteristics were balanced between the two treatment groups (Table 1).

**Table 1:** Characteristics of the participants at baseline.

	Total (n = 54)	Liraglutide (n = 31)	Placebo (n = 23)	P value
Sex (woman)	7 (13.0%)	2 (6.5%)	5 (21.7%)	0.12
Age (years)	66.8 (8.4)	66.7 (9.0)	66.8 (7.7)	0.96
Body mass index (kg/m^2^)	30.2 (4.9)	30.5 (5.3)	29.7 (4.3)	0.58
**Type 2 diabetes**				
Known duration in years	11.2 [5.2–19.8]	10.9 [4.7–19.8]	11.4 [5.2–21.3]	0.96
HbA_1c_ (mmol/mol)	57.4 (9.7)	56.9 (8.9)	57.9 (10.8)	0.72
**Kidney function**				
Estimated glomerular filtration rate (mL/min/1.73m^2^)	84.0 (17.6)	82.5 (19.3)	86.1 (15.1)	0.46
Urinary albumin creatinine rate (mg/g)	5.5 [4.5–15.5]	5.5 [4.5–15.5]	5.5 [4.0–16.0]	0.32
**Cardiovascular risk factors**				
Systolic blood pressure (mm Hg)	134 (16)	134 (16)	135 (17)	0.72
LDL cholesterol (mmol/L)	2.3 (0.68)	2.2 (0.67)	2.4 (0.71)	0.38
Current smoker	9 (16.7%)	7 (22.6%)	2 (8.7%)	0.27
HsCRP (mg/L)	2.3 [0.97–4.7]	1.7 [1.0–4.9]	2.7 [0.9–4.7]	0.33
History of cardiovascular disease^a^	11 (20.4%)	7 (22.6%)	4 (17.4%)	0.74
**Glucose lowering medication**				
Insulin use	19 (35.2%)	12 (38.7%)	7 (30.4%)	0.58
SGLT2 inhibitors	10 (18.5%)	4 (12.9%)	6 (26.1%)	0.29
**Cardiovascular medication**				
Aspirin treatment	19 (35.2%)	9 (29.0%)	10 (43.5%)	0.27
Lipid-lowering treatment	48 (88.9%)	29 (93.5%)	19 (82.6%)	0.38

Data are n (%), mean (SD) or median [IQR]. Differences in baseline characteristics between the liraglutide and the placebo group were tested using un-paired t-test and the χ^2^ test.

*hsCRP* high sensitivity C reactive protein, *SGLT2* sodium glucose transporter 2.

^a^A history of cardiovascular atherosclerotic disease was defined as a history of acute myocardial infarction, percutaneous coronary intervention, coronary artery bypass graft, stroke, peripheral arterial thrombosis, claudication and/or nitroglycerin requiring angina pectoris.

### Effects of treatment with liraglutide on inflammatory genes

We initially screened a small subset (n = 20) of the PBMC samples for expression of 12 inflammatory/anti-inflammatory genes: tumor necrosis factor-α (*TNFA*), interleukin 1β (*IL1B*), interferon-γ (*IFNG*), vascular cell adhesion molecule 1 (*VCAM1*), intercellular adhesion molecule 1 (*ICAM1*), C-X-C motif chemokine ligand 10 (*CXCL10*), interleukin 4 (*IL4*), interleukin 6 (*IL6*), interleukin 10 (*IL10*), interleukin 12 (*IL12*), cluster of differentiation 163 (*CD163*) and C–C Motif Chemokine Ligand 5 (*CCL5*) by quantitative real-time PCR. Of these, we detected measurable expression levels of the following six inflammatory genes: *TNFA, IL1B, ICAM, IFNG, CD163* and *CCL5*. The rest were either expressed at very low levels (Ct values > 38) or not detectable at all (data not shown). The six detectable inflammatory genes were then analyzed in the total population (n = 54).

**Figure 1:**
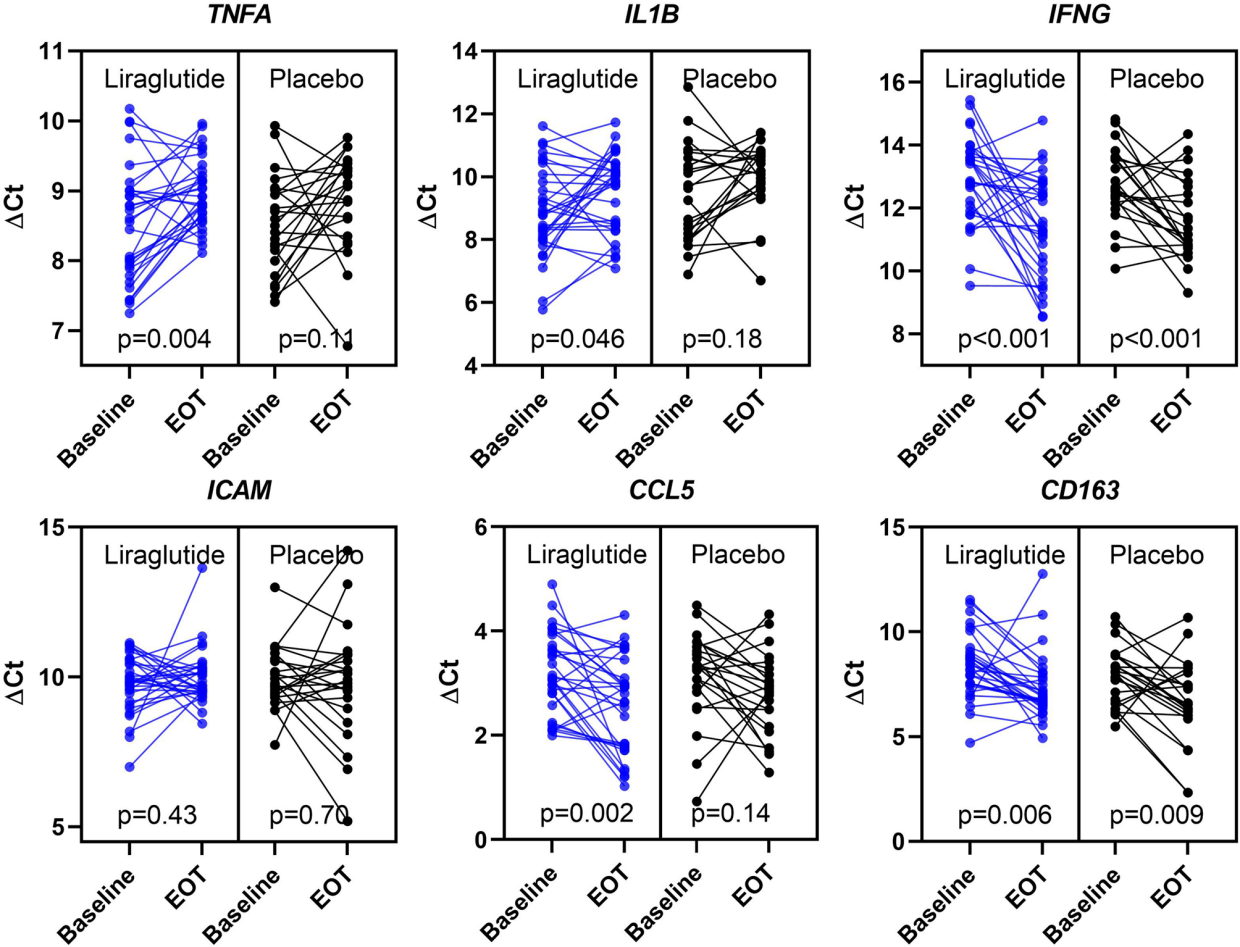
Effects of 26 weeks of treatment with liraglutide or placebo on the expression of inflammatory genes in isolated peripheral blood mononuclear cells in patients with T2D. Gene expression of selected inflammatory genes at baseline and end-of treatment measured by quantitative real-time polymerase chain reaction. Levels are for the individual participants in the liraglutide (n = 31) and the placebo (n = 23) treated group. *Ct* cycle threshold. ∆Ct = Ct (gene of interest) – Ct (*ACTB*), a decrease in ∆Ct values can be interpreted as an upregulation of the gene. P values from paired t-test. *EOT* end-of-treatment.

The expression of *TNFA* and *IL1B* was downregulated by 26% (p = 0.004) and 34% (p = 0.046), respectively, in the liraglutide-treated group from baseline to end-of-treatment. In the placebo group, the expression of *TNFA* and *IL1B* did not statistically change from baseline to end-of-treatment (p ≥ 0.11). However, there was no statistically significant differences, between the two groups (p ≥ 0.67, Table 2, Fig. 1).

**Table 2:** Changes in expression of selected inflammatory genes in PBMCs from 54 patients with T2D randomized to liraglutide vs placebo treatment.

	∆Ct			∆∆Ct		
	Baseline (SD)	End-of-treatment (SD)	P	Change (95% CI)	Fold change	P-value
***TNFA***						
Liraglutide n = 31	8.5 (0.82)	9.0 (0.49)	0.004	0.43 (0.15; 0.72)	0.74	0.67
Placebo n = 23	8.5 (0.70)	8.9 (0.70)	0.11	0.33 (-0.09; 0.75)	0.80	
***IL1B***						
Liraglutide n = 31	8.9 (1.4)	9.5 (1.3)	0.046	0.59 (0.01; 1.2)	0.66	0.83
Placebo n = 23	9.5 (1.5)	10.0 (1.1)	0.18	0.49 (-0.25; 1.2)	0.71	
***IFNG***						
Liraglutide n = 31	12.9 (1.4)	11.4 (1.6)	0.0002	-1.5 (-2.3; -0.77)	2.8	0.47
Placebo n = 21	12.7 (1.2)	11.6 (1.3)	0.002	-1.1 (-1.8; -0.46)	2.1	
***ICAM1***						
Liraglutide n = 31	9.8 (0.97)	10.0 (0.93)	0.43	0.22 (-0.34; 0.78)	0.86	0.43
Placebo n = 21	10.0 (1.0)	9.8 (1.9)	0.70	-0.16 (-1.0; 0.69)	1.1	
***CD163***						
Liraglutide n = 30	8.4 (1.6)	7.3 (1.6)	0.006	-1.1 (-1.8; -0.34)	2.1	0.74
Placebo n = 23	7.9 (1.4)	6.7 (2.0)	0.009	-1.3 (-2.2; -0.35)	2.5	
***CCL5***						
Liraglutide n = 30	3.2 (0.79)	2.6 (0.92)	0.002	-0.61 (-0.98; -0.24)	1.5	0.36
Placebo, n = 23	3.1 (0.87)	2.8 (0.79)	0.14	-0.35 (-0.81; 0.12)	1.3	

Gene expression of selected inflammatory genes before and after 26-weeks of treatment with liraglutide vs placebo measured by quantitative real-time PCR. *Ct* cycle threshold. ∆Ct = Ct (gene of interest) – Ct (*ACTB)*. ∆∆Ct = ∆Ct (baseline) − ∆Ct (end-of-treatment). A positive ∆∆Ct is interpreted as an upregulation of the gene, a negative ∆∆Ct is interpreted as a downregulation of the gene. Fold change = 2^−∆∆Ct^. Data are mean (SD) or change (95% CI). Paired t-test for comparisons between baseline and end-of-treatment within groups and unpaired t-test for comparison of the change from baseline to end-of-treatment between the two groups.

The expression of *IFNG* and *CD163* was upregulated in both the liraglutide (p < 0.006) and the placebo (p < 0.009) treated groups, with no statistically significant differences between the two groups (p ≥ 0.47).

The expression of *CCL5* was upregulated by 50% in the liraglutide-treated group (p = 0.002), whereas *CCL5* remained unchanged in the placebo group (p = 0.14). The difference between the two groups was not significant (p = 0.36).

The expression of *ICAM* was unchanged in both the liraglutide and the placebo-treated groups (p ≥ 0.43).

As GLP-1 RA treatment could affect PBMC inflammatory gene expression either directly via activating GLP-1 receptors (GLP-1R) at the surface of the PBMCs or indirectly via other phenotype changes (e.g. improved glycemic control and/or weight-reduction), we examined the expression of *GLP1R* in a subset (n = 40) of the PBMC samples to assess if liraglutide exert direct effects through GLP-1R on the PBMCs. In more than 90% of the samples examined, we could not detect *GLP1R* expression defined as no measurable expression or a Ct value ≥ 39 (data not shown). In positive control samples (pancreatic islets), the Ct values for *GLP1R* were around 31 (data not shown).

### Effects of GLP-1 on LPS-induced inflammatory gene expression in THP-1 cells

**Figure 2:**
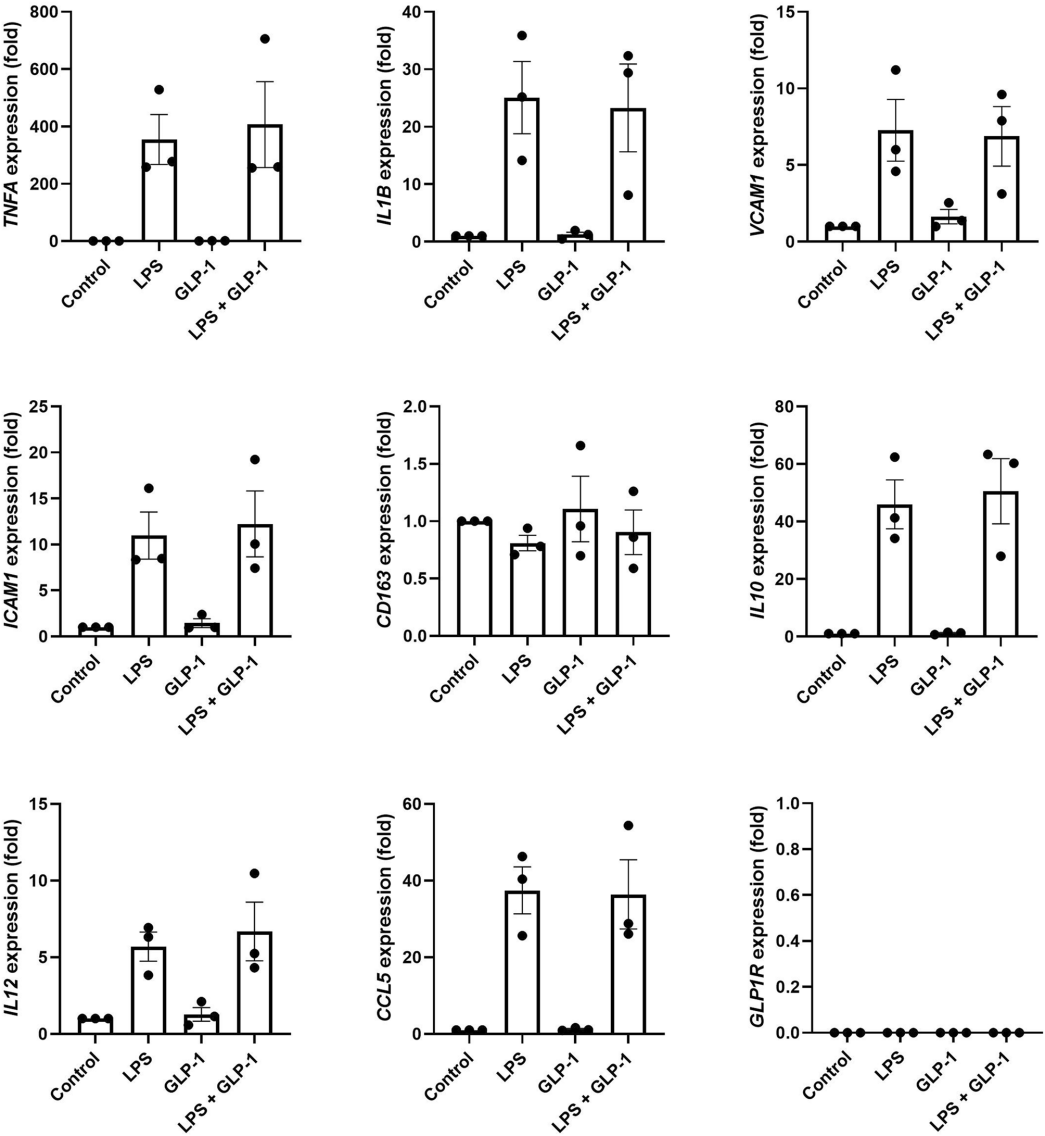
Effects of GLP-1 on the expression of inflammatory genes in human THP-1 cells. THP-1 cells were left untreated or exposed to 5 ng/ml LPS for 3 h in the presence or absence of 2.5 nM GLP-1. The expression of selected inflammatory genes and *GLP1R* was determined by real-time PCR. Gene expression was normalized to that of a housekeeping gene (*ACTB*) and data are presented as means ± SEM of 3 individual experiments. *GLP-1* glucagon-like peptide 1, *LPS* lipopolysaccharide.

Human THP-1 cells are monocyte-like immortalized cells derived from peripheral blood and constitute a widely used model of primary human monocytes^26^. We used THP-1 cells to further examine the potential direct effects of GLP-1 receptor activation on inflammatory genes in in vitro experiments. THP-1 cells were exposed to LPS to induce inflammatory gene expression in the presence or absence of 2.5 nM recombinant GLP-1. GLP-1 did not affect the expression of any of the examined genes (Fig. 2) suggesting that GLP-1 does not exert a direct effect on the monocytes. In accordance with this and our findings on PBMCs, the expression of *GLP1R* was also not detectable in THP-1 cells (Fig. 2).

## Discussion

In a sub-study of a randomized clinical trial including 54 participants with T2D treated for 26 weeks with liraglutide or placebo, we observed that liraglutide by end-of-treatment significantly decreased the expression of *TNFA* and *IL1B,* and upregulated *CCL5* in PBMCs as compared to baseline. These effects were not seen in the placebo group. However, statistical significance was lost when comparing the liraglutide and placebo treated groups.

Overall, our data supports a discrete anti-inflammatory effect of GLP-1 RA in patients with T2D which is in accordance with previous studies. Hence, results from in vitro experiments, preclinical studies and small clinical studies have suggested anti-inflammatory effects of GLP-1 RA^15,16,27^. Specifically, the observation that one year of liraglutide treatment reduces plasma hsCRP by 35% (from a baseline level of 3.9 mg/L) in a randomized clinical trial in 3,731 obese patients without T2D, evidently shows that GLP-1 RA treatment has anti-inflammatory effects^17^.

The mechanisms linking GLP-1 RA treatment to anti-inflammatory actions in distinct organs and tissues remain uncertain. GLP-1 RA treatment reduces weight and improves glycemic control^28,29^ and could modulate inflammation indirectly through associated weight-loss or improved glycemic control, or alternatively by directly targeting GLP-1R expressed in multiple human organs and cells. It has been proposed that a GLP-1 RA could directly target GLP-1R on populations of circulating immune cells, as a possible mechanism to modulate inflammation in peripheral organs^27^. However, as pointed out by Drucker et al., definitive identification of GLP-1Rs in immune cell populations is often lacking and direct effects of GLP-1 RAs on immune cells remain controversial^27^. We find that PBMCs, seen as one cell population, from patients with T2D do not express the *GLP-1R* at the mRNA level, and confirmatory we observed that human THP-1 monocytes also do not express *GLP-1R*. These findings indicate that an effect of GLP-1 RAs on PBMCs most likely would be a secondary effect to other GLP-1 RA effects exerted in other tissues and/or a consequence of phenotypic changes such as weight loss or improved glycemic control. It is worth noticing that although THP-1 cells are generally considered a good model of primary monocytes, there are limitations with the use of these cells. For example, THP-1 cells are less responsive to LPS than primary human monocytes which may partly be due to lower expression of CD14^26^. In some respects, THP-1 cells may therefore be a poor model of primary monocytes and results should therefore be interpreted with caution. Further, as PBMCs consist of several immune cell types, it cannot be excluded that some of the sub-cell types in the PBMC fraction consistently express *GLP1R,* but this expression becomes undetectable when analyzing the PBMCs as one cell population. In favor of a possible direct effect via GLP-1R on immune cells is the finding that the supernatants from PBMCs isolated from 10 patients with recent-onset T2D and incubated in vitro for 24 h with exenatide showed reduced levels of TNFα, IL-1β, IL-6 and CCL5^30^. However, these findings need confirmation and further investigations are required to unravel if GLP-1 RA treatment has direct effects on immune cells through GLP-1R activation.

We report downregulated expression of *TNFA* and *IL1B* in the group treated with liraglutide, however, these changes lost significance when compared to placebo. In support of a modest anti-inflammatory effect of liraglutide, we have previously reported how circulating markers of inflammation, including hsCRP, TNF-α and ICAM-1 were unchanged by liraglutide treatment in the 102 participants included in the main trial^19^. Few small clinical studies have previously evaluated the effect of GLP-1 RA treatment on expression of inflammatory genes in PBMCs. In a prospective study Savchenko et al. gave liraglutide (1.2 mg daily) to 15 obese patients with T2D for 6 weeks and observed a reduced mRNA expression of *TNFA* in PBMCs^12^. Chaudhuri et al. demonstrated that in 24 obese persons with T2D, 12 weeks of treatment with the GLP-1 RA exenatide reduced PBMC mRNA expression of *TNFA* and *IL1B* compared to placebo^18^. These reported changes in mRNA expression of *TNFA* and *IL1B* could be due to direct effects of exenatide and/or be secondary to metabolic improvements in a clinical setting, as the authors reported significant reductions in HbA_1c_, free fatty acids and insulin, while body weight was unchanged.

### Strengths and limitations

Our study has the strengths that the original study was a double-blind randomized placebo controlled trial and we used liraglutide, a potent GLP-1 RA, which opposed to exenatide^31^, has established cardiovascular benefits observed in outcome studies^3^. Further, our study is large, and compared to the randomised study using exanatide by Chaudhuri et al.^18^ we included more than double the number of patients and our intervention period was twice as long. We acknowledge that our study has important limitations, including that it was not designed with the primary aim of investigating effects of liraglutide on PBMC gene expression and therefore the original randomization was not aimed for this exploratory sub-study. Although randomly selected, the two groups (liraglutide vs placebo) appear numerically unbalanced for possible confounding factors in the assessment of inflammatory gene expression including the percentage of women (numerically lower in the liraglutide group), the percentage of smokers (numerically higher in the liraglutide group), and the use of SGLT-2 inhibitors with a favorable impact on cardiovascular outcomes^32^ and possibly inflammation (numerically lower in the liraglutide group). The distribution of these characteristics, however, was not statistically significant between the liraglutide and the placebo treated groups but we cannot rule out that these differences might have impacted our results. Finally, by random chance, we ended up with a skewed distribution (31 vs 23) of samples from the liraglutide and the placebo groups.

## Conclusions

In a sub-study of a randomized clinical trial in patients with T2D treated for 26 weeks with liraglutide, we observed a discrete modulatory effect of liraglutide on the expression of inflammatory genes in PBMCs. The lack of evidence for *GLP1R* expression in PBMCs and THP-1 cells, suggests that possible effects of liraglutide on the PBMCs’ gene expression are most likely indirect. Further investigations are needed to establish the link between the anti-inflammatory potential and cardio-protective effect of GLP-1 RA and if GLP-1 RAs have direct anti-inflammatory effects on immune cells.

## Data Availability

Access to the data used to support the findings of this study are restricted by the Danish regional scientific ethical committees in order to protect patient privacy. Data are available upon reasonable request for researchers who meet the criteria for access to confidential data.

## References

[1] van Greevenbroek MM, Schalkwijk CG, Stehouwer CD (2013). Obesity-associated low-grade inflammation in type 2 diabetes mellitus: Causes and consequences. Neth. J. Med..

[2] Lopez-Candales A, Hernandez Burgos PM, Hernandez-Suarez DF, Harris D (2017). Linking chronic inflammation with cardiovascular disease: from normal aging to the metabolic syndrome. J. Nat. Sci..

[3] Marso SP (2016). Liraglutide and cardiovascular outcomes in type 2 diabetes. N. Engl. J. Med..

[4] Marso SP (2016). Semaglutide and cardiovascular outcomes in patients with type 2 diabetes. N. Engl. J. Med..

[5] Hernandez AF (2018). Albiglutide and cardiovascular outcomes in patients with type 2 diabetes and cardiovascular disease (harmony outcomes): A double-blind, randomised placebo-controlled trial. Lancet.

[6] Gerstein HC (2019). Dulaglutide and cardiovascular outcomes in type 2 diabetes (REWIND): A double-blind, randomised placebo-controlled trial. Lancet.

[7] Rizzo M (1864). GLP-1 receptor agonists and reduction of cardiometabolic risk: Potential underlying mechanisms. Biochim. Biophys. Acta Mol. Basis Dis..

[8] Nikolic D (2021). Liraglutide reduces carotid intima-media thickness by reducing small dense low-density lipoproteins in a real-world setting of patients with type 2 diabetes: A novel anti-atherogenic effect. Diabetes Ther..

[9] Rizzo M (2016). Liraglutide improves metabolic parameters and carotid intima-media thickness in diabetic patients with the metabolic syndrome: An 18-month prospective study. Cardiovasc. Diabetol..

[10] Giglio, R. V. *et al.* Liraglutide increases serum levels of microRNA-27b, -130a and -210 in patients with type 2 diabetes mellitus: A novel epigenetic effect. *Metabolites ***10**. 10.3390/metabo10100391 (2020). 10.3390/metabo10100391 PMC7599907 33008044

[11] Rizzo M (2015). Liraglutide reduces oxidative stress and restores heme oxygenase-1 and ghrelin levels in patients with type 2 diabetes: A prospective pilot study. J. Clin. Endocrinol. Metab..

[12] Savchenko LG (2019). Liraglutide exerts an anti-inflammatory action in obese patients with type 2 diabetes. Rom. J. Intern. Med..

[13] Diaz-Soto G (2014). Beneficial effects of liraglutide on adipocytokines, insulin sensitivity parameters and cardiovascular risk biomarkers in patients with type 2 diabetes: A prospective study. Diabetes Res. Clin. Pract..

[14] Patti, A. M. *et al.* Impact of glucose-lowering medications on cardiovascular and metabolic risk in type 2 diabetes. *J. Clin. Med*. **9**, 912. 10.3390/jcm9040912 (2020). 10.3390/jcm9040912 PMC7230245 32225082

[15] von Scholten BJ (2017). Effects of liraglutide on cardiovascular risk biomarkers in patients with type 2 diabetes and albuminuria: A sub-analysis of a randomized, placebo-controlled, double-blind, crossover trial. Diabetes Obes. Metab..

[16] Hogan AE (2014). Glucagon-like peptide 1 analogue therapy directly modulates innate immune-mediated inflammation in individuals with type 2 diabetes mellitus. Diabetologia.

[17] Pi-Sunyer, X. *et al.* A randomized, controlled trial of 3.0 mg of liraglutide in weight management. *N. Engl. J. Med.* **373**, 11–22. 10.1056/NEJMoa1411892 (2015). 10.1056/NEJMoa1411892 26132939

[18] Chaudhuri A (2012). Exenatide exerts a potent antiinflammatory effect. J. Clin. Endocrinol. Metab..

[19] Ripa, R. S. *et al.* Effect of liraglutide on arterial inflammation assessed as [(18)F]FDG uptake in patients with type 2 diabetes: A randomized, double-blind, placebo-controlled trial. *Circ. Cardiovasc. Imaging*. doi:10.1161/CIRCIMAGING.120.012174 (2021). 10.1161/CIRCIMAGING.120.012174 PMC8300846 34187185

[20] Jacobsen LV, Flint A, Olsen AK, Ingwersen SH (2016). Liraglutide in type 2 diabetes mellitus: Clinical pharmacokinetics and pharmacodynamics. Clin. Pharmacokinet..

[21] Barale C (2017). Glucagon-like peptide 1-related peptides increase nitric oxide effects to reduce platelet activation. Thromb. Haemost..

[22] Reiner DJ (2016). Astrocytes regulate GLP-1 receptor-mediated effects on energy balance. J. Neurosci..

[23] Cheng YH, Ho MS, Huang WT, Chou YT, King K (2015). Modulation of glucagon-like peptide-1 (GLP-1) potency by endocannabinoid-like lipids represents a novel mode of regulating GLP-1 receptor signaling. J. Biol. Chem..

[24] Chen D (2007). A nonpeptidic agonist of glucagon-like peptide 1 receptors with efficacy in diabetic db/db mice. Proc. Natl. Acad. Sci. U S A.

[25] Fan H (2015). The non-peptide GLP-1 receptor agonist WB4-24 blocks inflammatory nociception by stimulating beta-endorphin release from spinal microglia. Br. J. Pharmacol..

[26] Bosshart H, Heinzelmann M (2016). THP-1 cells as a model for human monocytes. Ann. Transl. Med..

[27] Drucker DJ (2016). The cardiovascular biology of glucagon-like peptide-1. Cell Metab..

[28] Mirabelli, M. *et al.* Long-term effectiveness of liraglutide for weight management and glycemic control in type 2 diabetes. *Int. J. Environ. Res. Public Health*. 10.3390/ijerph17010207 (2019). 10.3390/ijerph17010207 PMC6981922 31892206

[29] Mirabelli, M. *et al.* Clinical effectiveness and safety of once-weekly GLP-1 receptor agonist dulaglutide as add-on to metformin or metformin plus insulin secretagogues in obesity and type 2 diabetes. *J. Clin. Med.*10.3390/jcm10050985 (2021). 10.3390/jcm10050985 PMC7957905 33801192

[30] He L (2013). Anti-inflammatory effects of exendin-4, a glucagon-like peptide-1 analog, on human peripheral lymphocytes in patients with type 2 diabetes. J. Diabetes Investig..

[31] Holman RR (2017). Effects of once-weekly exenatide on cardiovascular outcomes in type 2 diabetes. N. Engl. J. Med..

[32] Zinman B (2015). Empagliflozin, cardiovascular outcomes, and mortality in type 2 diabetes. N. Engl. J. Med..

